# Role of Psychosomatic Symptoms in COVID-19 Vaccine Hesitancy

**DOI:** 10.3390/vaccines11050922

**Published:** 2023-04-30

**Authors:** Saral Desai, Tejasvi Kainth, Garima Yadav, Hansini Kochhar, Sushma Srinivas, Saher Kamil, Wei Du

**Affiliations:** 1 Department of Psychiatry, Tower Health-Phoenixville Hospital, Phoenixville, PA 19460, USA; 2 Department of Psychiatry, Stony Brook University, Stony Brook, NY 11794, USA; 3 Department of Psychiatry, Bronx Care Health System, Bronx, NY 10456, USA; 4 Department of Psychiatry, Maimonides Medical Center, Brooklyn, NY 11219, USA; 5 Department of Psychiatry, A.J. Institute of Medical Sciences and Research Center, Manglore 575004, India; 6 Department of Pediatrics, Dell Children’s Medical Center, Austin, TX 78723, USA; 7 Academic Affairs, Tower Health, West Reading, PA 19611, USA; 8 Department of Psychiatry, Drexel University College of Medicine, Philadelphia, PA 19102, USA

**Keywords:** COVID-19 vaccine, vaccine hesitancy, psychosomatic symptoms, nocebo effects, immunization, health behavior

## Abstract

Vaccination against COVID-19 is one of the highly effective preventative strategies to reduce morbidity and mortality associated with COVID-19 infection. The rapid approval of COVID-19 vaccination due to the raging pandemic, media coverage, anti-vaccination groups, and concerns about adverse effects associated with vaccination has given rise to COVID-19 vaccine hesitancy. Current evidence suggests that psychosomatic and nocebo-related adverse effects account for a significant proportion of common adverse effects following COVID-19 vaccination. The most common adverse effects are headache, fatigue, and myalgia, which are highly prone to nocebo effects. In our review article, we discuss the role of psychosomatic and nocebo effects in COVID-19 vaccination-related hesitancy, predictors of such effects, and strategies to reduce vaccine hesitancy. General education regarding psychosomatic and nocebo effects and specialized education for at-risk populations may reduce psychosomatic and nocebo-related adverse effects following COVID-19 vaccination, ultimately reducing hesitancy.

## 1. Introduction

In March 2020, the World Health Organization (WHO) declared yet another pandemic, with confirmed cases of COVID-19 exceeding 600 million (632,533,408), including over six million (6,592,320) deaths reported as of November 2022 [1]. This pandemic has had a profound global impact on both physical and mental health, livelihoods, education, and the economy as a whole.

Severe acute respiratory syndrome coronavirus 2 (SARS-CoV-2) is a strain of coronavirus that causes COVID-19 disease. Although social distancing has been an effective measure to reduce the spread of SARS-CoV-2, vaccination remains the most effective long-term solution to combat this pandemic, as demonstrated during prior infectious pandemics like influenza. Since the onset of the COVID-19 pandemic, the FDA has authorized or approved four vaccines for the prevention of COVID-19. Pfizer-BioNTech’s and Moderna’s COVID-19 Vaccines are mRNA vaccines that use genetically engineered RNA to generate a protein that stimulates an immune response. Janssen’s COVID-19 vaccine is developed using viral vector technology, in which a modified adenovirus delivers the genetic code for the SARS-CoV-2 antigen into cells to trigger immune responses. AstraZeneca, Reithera, and Sputnik have also developed their vaccines using the same technology. Novavax COVID-19 vaccine contains pieces of the inactivated or weakened virus (proteins) specifically the spike protein which then triggers immune responses once administered.

According to COVID-19 Epidemiology and Vaccination Rates reported by the CDC in July 2022, approximately 26–37 million (13.9%) adults in the United States had not yet received a COVID-19 vaccine [2,3]. The Emergency Use Authorization and accelerated approval of vaccines against COVID-19, in response to the pandemic without longitudinal safety data, potential adverse effects, and long-term investigations, have raised concerns and led to anxiety and vaccine hesitancy [4]. Vaccine-related side effects, such as pain at the injection site, fatigue, headache, chills, fever, and nausea, have caused some in the general public to remain unvaccinated. Vaccine-related myths and misinformation in the community regarding infertility, poisoning, and other non-evidence-based serious side effects have fueled anxiety and vaccine avoidance among some members of the community.

The efficacy of a vaccine is dependent on both the characteristics of the vaccine itself and the individual receiving it. Research has indicated that psychological factors play a crucial role in the immune response to vaccines [5]. In particular, stress, depression, loneliness, and poor health behaviors, particularly in vulnerable populations, may contribute to the prevalence and severity of vaccine-related side effects. Despite widespread distribution and administration of vaccines among large populations, vaccine hesitancy continues to persist. Concerns regarding vaccine efficacy and associated side effects have led to an increased incidence of vaccine-related side effects due to anxiety and stress [5].

In this review, we describe the critical role that psychosomatic symptoms play in vaccine hesitancy. We differentiate psychosomatic symptoms due to anxiety, fear, and perceived expectations of vaccine side effects from the symptoms caused by COVID-19 vaccine ingredients and the inflammatory protective response it elicits. We also discuss the role of anxiety and comorbid psychiatric disorders in experiencing psychosomatic symptoms, as well as the implications and measures to combat vaccine hesitancy by recognizing and addressing psychosomatic symptoms after COVID-19 vaccination.

## 2. Discussion

### 2.1. Psychosomatic Symptoms and Somatic Symptom Disorders

Psychosomatic symptoms refer to physical symptoms that are believed to be caused or exacerbated by psychological factors [6]. These symptoms can affect any part of the body and range from mild to severe. Although the exact cause of psychosomatic symptoms is not always clear, it is believed that psychological factors such as stress, anxiety, and depression play a significant role. Some of the most common psychosomatic symptoms include headaches, stomach pains, chest pains, and fatigue [7]. Psychosomatic symptoms are also recognized and defined by the Diagnostic and Statistical Manual of Mental Disorders, Fifth Edition (DSM-5). The DSM-5 refers to psychosomatic symptoms as Somatic Symptom Disorder (SSD), which is characterized by persistent and distressing physical symptoms that may or may not have a known medical cause. These symptoms often lead to excessive thoughts, feelings, and behaviors related to the symptoms and can significantly impact an individual’s quality of life. The DSM-5 criteria for SSD includes the presence of one or more somatic symptoms that cause significant distress or impairment, excessive and disproportionate thoughts, feelings, or behaviors related to the symptoms, and symptoms lasting for at least six months [8].

### 2.2. Psychosomatic Symptoms in COVID-19 Vaccination

#### 2.2.1. COVID-19 Vaccination and Nocebo Effect

Nocebo effects refer to negative effects that occur after the administration of an inactive or harmless substance due to the patient’s negative expectations and beliefs about the treatment [9]. The current evidence suggests that nocebo effects are highly prevalent in recipients of COVID-19 vaccinations. For example, in a recent systematic review and meta-analysis, which included reports of adverse effects in 45,380 participants, it was found that 35% of those who received a placebo experienced systemic negative effects after the first dose, and 32% experienced the same after the second [10]. The vaccine groups reported more negative effects, but it was observed that the placebo arms (“nocebo responses”) accounted for a large percentage of the negative effects after the first dose (76%) and the second dose (52%) [10]. The most common negative effects reported in the placebo groups were headache and fatigue, experienced by 19.3% and 16.7% of participants after the first dose, respectively [10]. Another systematic review of randomized control trials found similar results that the majority of common adverse effects associated with COVID-19 vaccines, such as fatigue, headache, and myalgia, may be related to the nocebo effect [11].

#### 2.2.2. Relationship between Nocebo Effects and Psychosomatic Symptoms

The most common nocebo responses that accounted for adverse effects following COVID-19 vaccination were headache and fatigue. These are perhaps also the most commonly reported somatic symptoms in patients suffering from somatic symptom disorder.

People who are prone to psychosomatic symptoms may be more susceptible to nocebo effects [12]. This is because individuals who experience psychosomatic symptoms are more likely to be sensitive to physical sensations and pay close attention to changes in their bodies. This heightened sensitivity can make them more aware of negative side effects or symptoms that may be caused by the expectation of harm [13]. Another way in which psychosomatic symptoms and nocebo effects may be related is through the role of negative expectations. People who experience psychosomatic symptoms may be more likely to have negative expectations or beliefs about their health, which might increase their susceptibility to nocebo effects. While limited research exists on the topic, it seems that there may be a complex interplay between psychological factors, physical symptoms, and the expectation of harm.

#### 2.2.3. Psychosomatic Symptoms vs. Vaccine-Induced Side Effects

There are two types of reactions to vaccines whenever they are administered: (a) those that are attributed to the ingredients in the vaccine and (b) those that are not caused by the vaccine itself but occur due to the stress and stress response of getting immunized. Adverse reactions are often misinterpreted in both social and news media, with immunization stress reactions being presented as evidence of vaccines’ harmfulness [14].

It is essential to understand these nocebo effects that occur when there is a negative expectation about a treatment or intervention, such as a vaccine, as it causes the person to experience side effects unrelated to the vaccine [15]. These effects can range from nausea, headache, pain at the injection site, cognitive ability perception, cardiovascular function, numbness, tingling and agitation, tics, and twitches [16].

People with high levels of somatic perception, fear of pain, and high levels of negative emotionality (i.e., neuroticism) and those who tend to misinterpret harmless body sensations as adverse effects are more prone to have nocebo reactions to vaccines or treatments [16]. It has been reported that nocebo effects are commonly experienced by people with a history of mood disorders, anxiety disorders, or medically unexplainable physical symptoms [17].

In 2018, the WHO [18] reported that certain negative events could occur following vaccination. These nocebo effects are generally immunization stress-related responses (ISRRs) triggered by the vaccination process rather than vaccine components. There are four groups of reactions in the ISRRs: (1) stress response and acute anxiety, (2) mass psychogenic illness, (3) vasovagal reactions, and (4) functional neurological disorders. These nocebo reactions can occur before, during, or after immunization.

The most common anxiety and stress-related responses are headaches, palpitations, faintness, dizziness, and stress-related hyperventilation [18]. Additionally, anxiety or stress-related symptoms may be amplified due to the immunization process. Studies have shown that vaccination-related stress and anxiety can manifest as psychosomatic symptoms, including headaches, muscle tension, and gastrointestinal disturbances [14,15]. These reactions are commonly seen in cohesive social settings, schools, or other closed settings [19]. Vasovagal reactions involving injection are tachycardia followed by bradycardia, faintness, and syncope. It can also occur due to blood-injury-reaction phobia [8], and syncope often occurs immediately or within 30 min after vaccination [18].

Symptoms or bodily reactions resembling organic illnesses, but without an identified cause, are known as Mass Psychogenic Illnesses (MPI) [20]. This phenomenon can occur in people with shared beliefs about the cause of the symptoms. The symptoms, spreading mechanism, ripple effects, and demographics of vaccine-related MPI are similar to other forms of MPI, with children and adolescents being the most affected [20]. In addition to headaches, dizziness, weakness, nausea, trembling, and fainting, there may also be functional neurological symptoms such as difficulty walking or speaking, or pseudo-seizures [20].

Like with MPI in general, vaccine-related MPI symptoms usually develop rapidly with an acute onset, spread quickly, and resolve promptly, often within hours or days following assurance given to the affected individuals (and their parents in the case of minors). Functional neurological disorders (FNDs), previously referred to as conversion disorders, are mental health conditions that are characterized by one or more motor or sensory reactions that appear to be neurological symptoms but are not consistent with any known neurological diseases [8]. FNDs can result from acute physiological reactions to immunization, including vasovagal responses, localized pain, and flu-like symptoms [21]. Several cases of FNDs have been reported during COVID-19 pandemic [22,23]. A combination of physical rehabilitation, cognitive-behavioral therapy, and education can help treat FND [23,24].

The side effects related to vaccine components differ from the nocebo effects. Any vaccine, including the COVID-19 vaccine, can cause common systemic or local reactions. Erythema, tenderness, and induration at the injection site, as well as occasional abscess formation, are categorized as local reactions. Systemic reactions, on the other hand, may include fever, headache, cough, coryza, and, in rare instances, anaphylactic reactions [25]. Notably, adverse events of special interest (AESI) are a particular type of negative reaction that can present as autoimmune diseases or affect different organs, including but not limited to the renal, dermatologic, hematologic, lymphatic, ocular, gastrointestinal, cardiovascular, and neurological systems. These types of adverse events are uncommon and warrant additional evaluation [26]. Autoimmune complications reported to occur with the COVID-19 vaccine include acquired hemophilia A, immune-mediated thrombocytopenia (ITP), anaphylaxis, capillary leak syndrome, cutaneous vasculitis, IgA vasculitis, thyroiditis, IgA nephropathy, acute macular neuroretinopathy, bilateral retinal detachment, uveitis, and pulmonary thromboembolism [27]. Further investigation is necessary to establish a cause-and-effect relationship as there is currently no substantial evidence suggesting that COVID-19 vaccination is primarily responsible. However, since these adverse events can be life-threatening, monitoring them is essential [27].

After receiving the COVID-19 vaccine, various neuropsychiatric symptoms have been reported and are thought to be associated with various potential mechanisms, including dysregulated immunomodulation [28]. These neuropsychiatric manifestations include acute confusional states (altered mental status), psychosis, affective disorders (mania and depression), and functional neurological disorders [29]. Most of these cases involved young and middle-aged adults, and both sexes were equally affected. There is a need to further research and collect data about the psychiatric adverse effects of the COVID-19 vaccine [29]. It is possible that psychosomatic experiences may have contributed to some of the reported adverse events.

#### 2.2.4. Predictors of Psychosomatic Symptoms following Vaccination

According to a US poll, 90% of people who were hesitant to receive the COVID-19 vaccination were more concerned about its side effects than the infection itself [30]. It has been suggested that hesitancy towards the COVID-19 vaccination and its associated side effects might be influenced by nocebo effects [31]. Nocebo-related effects and COVID-19 vaccination side effects have been linked by various factors, including expectations of side effects prior to receiving the vaccine, stress regarding COVID-19 infection, and depressive symptoms. This can help predict COVID-19 vaccine side effects (i.e., headache, fatigue, and pain at the injection site), and the prediction goes beyond baseline symptomatology, age, vaccine type, and prior COVID-19 infection [32]. Nocebo effects are also linked to long COVID-19 symptoms, as it has been observed that many individuals who reported 10–12 months of long COVID-19 illness had negative antibody test results, suggesting a lack of history of COVID-19 infection [33]. Modifiable nocebo-related factors that could potentially reduce vaccine hesitancy toward and side effects from COVID-19 vaccination include addressing negative expectations, reducing exposure to negative media coverage, and providing insightful education on nocebo effects [32].

#### 2.2.5. Psychiatric Comorbidities and Psychosomatic Symptoms following Vaccination

Psychosocial stressors associated with the COVID-19 pandemic, such as social isolation, lockdowns, financial burdens, and loss of jobs and family members, have had a very deleterious effect on mental health. Although there is no conclusive evidence, psychiatric comorbidities may be associated with COVID-19 vaccination in a very small population. Neuropsychiatric manifestations have been observed after the first dose and during the 0–10 days after vaccination. Although viral vector vaccines like the Oxford AstraZeneca vaccines were mostly associated with more psychiatric manifestations, novel vaccines that used relatively newer technology, like mRNA vaccines, were also associated with vaccine hesitancy, anxiety, and panic in users [34].

COVID-19 infection is associated with a range of neuropsychiatric manifestations, which are believed to be mediated by the virus’s direct effect on neurons, leading to the development of inflammation and neuropsychiatric symptoms [35]. Another theory suggests that the virus can trigger an exaggerated immune response, known as a cytokine storm, which can also lead to brain inflammation and subsequent neuropsychiatric symptoms [36]. Vaccination could also trigger a smaller but similar immune response, potentially leading to the development of neuropsychiatric symptoms [37]. Case reports have described delirium, psychosis, mania, depression, anxiety, and confusion, among other symptoms, in both psychiatric and non-psychiatric patients [29,38,39,40,41,42,43,44,45,46].

Wang et al. showed that preinfectional anxiety, depression, and loneliness were associated with an increased risk of developing post-COVID-19 conditions following infection [47]. A similar association might exist between preexisting poor mental health and the risk of adverse reactions following COVID-19 vaccination. However, the research on that specific topic is currently limited.

#### 2.2.6. Addressing Nocebo/Psychosomatic Symptoms to Reduce Vaccine Hesitancy Potentially

A perceived lack of understanding about COVID-19 and the COVID-19 vaccination is one of the main causes of vaccine aversion. As discussed earlier, nocebo effects and psychosomatic symptoms could potentially increase vaccine hesitancy. It is no surprise, then, that lower perceived safety of COVID-19 immunization was associated with a higher expectation of side effects [48,49]. Therefore, it is essential to communicate accurate information regarding the side effects of the vaccine, including the incidence and the moderate and transient nature of side effects that are most likely to be encountered by COVID-19 vaccination recipients.

Earlier, contextualized informed consent was proposed as a method to lower the nocebo reaction by allowing physicians to customize information about potential treatment adverse effects for the patient [50]. However, this method is thought to impede informed consent.

It is crucial to be transparent about what to expect. Although educational initiatives are promising, there is conflicting research on their impact on vaccination rates [51,52]. The accuracy of side-effect expectancies can be enhanced by using simple infographics (such as pictographs) and improving the clarity and readability of information [53,54].

Interestingly, psychological factors, notably expectations, influence the perception of side effects following immunization [48]. An approach that can prove to be useful is positive framing; for example, rather than telling patients that “a minority of patients experience side effects,” the message could be reframed as “the majority of people do not experience side effects” [55]. A study showed that participants who received a positive framing of the likelihood of side effects had fewer systemic side effects and lower work absenteeism [56]. This strategy ensures that patients have access to all pertinent information regarding a novel treatment (even when presented in a more positive light) to enable them to make an informed choice and reduce any potential negative effects of the nocebo effect. It is to be noted that as the quantity and variety of projected adverse effects grow, the utility of this strategy is likely to decline.

Placing emphasis on the possibility of not experiencing adverse effects may also be beneficial [57]. Although additional research on communication tactics is needed, such transparency contributes to full disclosure and is unlikely to cause further harm. Another approach proposed is alerting the public about the possibility of nocebo reactions, which may help minimize concerns regarding COVID-19 immunization and alleviate vaccination hesitancy [58,59]. Adopting this approach, the nocebo effect is explained in simple terms while providing examples from everyday life. It illustrates how giving patients information about the side effects of treatment could result in them experiencing those symptoms or other symptoms that would otherwise be attributed to the treatment [14].

**Figure 1:**
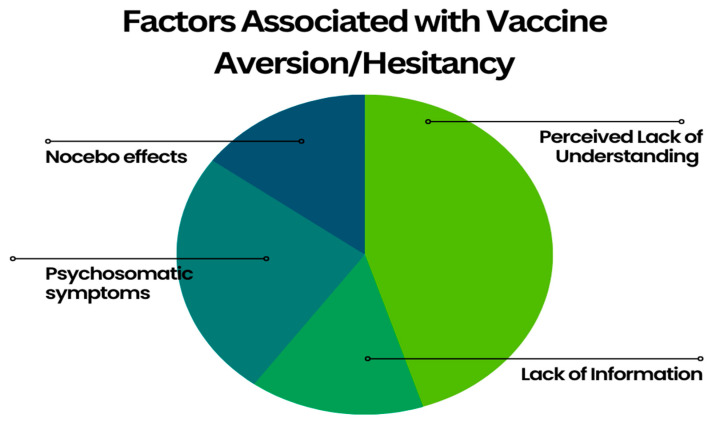
Factors Associated with Vaccine Aversion/Hesitancy.

Large groups of individuals getting vaccinated together, such as children and/or adolescents, are noted to be more easily triggered than the general public. Hence, vaccination clinics should be urged to vaccinate patients in cubicles or individual rooms that are out of direct sight of other patients. This method can eliminate the direct transmission of psychogenic stress reactions [60]. In conclusion, factors associated with vaccine aversion/hesitancy can be summarized in the Figure 1.

## 3. Conclusions

Psychosomatic symptoms and nocebo effect contribute significantly to many common COVID-19 vaccine-related adverse effects. Such adverse effects fuel COVID-19 vaccine hesitancy. General education regarding psychosomatic and nocebo effects and specialized education for at-risk populations may reduce psychosomatic and nocebo-related adverse effects following COVID-19 vaccination, ultimately reducing hesitancy.

## Data Availability

Data used for the review article are readily available with references included.
